# Myeloid derived suppressor cells in neuroblastoma: mechanisms of immune evasion and therapeutic opportunities

**DOI:** 10.3389/fimmu.2026.1773622

**Published:** 2026-03-26

**Authors:** Hae Soo Choo, Tina Yu, Timothy Pham, Neharika Singh, Shweta Joshi

**Affiliations:** 1 Department of Pediatrics, Division of Pediatric Hematology-Oncology, Moores Cancer Center, University of California, San Diego, CA, United States; 2 Division of Genomics and Precision Medicine, University of California, San Diego, CA, United States

**Keywords:** Immunosuppression, myeloid cells, myeloid derived suppressor cells, neuroblastoma, tumor microenvironment, tumor-associated macrophages

## Abstract

Neuroblastoma is the most common extracranial pediatric solid tumor. Although the incorporation of anti-GD2 immunotherapy into standard care has improved outcomes, five-year survival for high-risk patients remains below 50%. This highlights that, while immunotherapy holds promise in this pediatric cancer, neuroblastoma has developed multiple immunosuppressive mechanisms that limit anti-tumor immune responses. Among these, myeloid cells, including tumor associated macrophages (TAMs) and myeloid-derived suppressor cells (MDSCs) play a central role in promoting tumor progression and suppressing immune activity. MDSCs, which are primarily classified into monocytic (M-MDSC) and granulocytic (PMN-MDSC) subsets, are markedly increased in both murine neuroblastoma models and human patients, where they promote immunosuppression and impair T cell and NK cell functions. This review summarizes the myeloid landscape in neuroblastoma, covering the origin and development of MDSCs, the phenotypic and functional diversity of MDSC subsets, the mechanisms driving MDSC recruitment and immunosuppressive activity, and emerging therapeutic strategies to enhance immunotherapy efficacy, including approaches to target MDSCs and modulate ferroptosis to reprogram their function.

## 1 Introduction

Neuroblastoma, a pediatric tumor originating mainly in the sympathetic nervous system, displays significant clinical heterogeneity (1, 2). Patients are stratified into low-, intermediate-, and high-risk groups, with corresponding five-year survival rates of 90–95% for low- and intermediate-risk cases, and around 50% for high-risk disease (3). High-risk neuroblastoma is often associated with MYCN amplification, a key prognostic marker that correlates with aggressive tumor behavior and poor outcomes. For high-risk patients, treatment involves a multimodal approach, including chemotherapy, surgery, myeloablative chemotherapy with autologous stem cell transplantation, radiation therapy, and anti-disialoganglioside (GD2)–based immunotherapy (4). Anti-GD2 monoclonal antibody (mAb) therapy is well-tolerated in high-risk neuroblastoma and has been shown in a Phase III trial to improve event-free and overall survival when combined with retinoic acid, IL-2, and GM-CSF (5–7). However, the added benefit of IL-2 and GM-CSF remains unclear, as a recent Phase III trial showed no improvement with subcutaneous IL-2 (8). Consolidative therapy with anti-GD2 mAbs, such as dinutuximab, is now standard of care. Additionally, early-phase trials of GD2-targeted CAR-T cells demonstrate safety, feasibility, and encouraging preliminary results (9–14). However, due to immunosuppressive tumor microenvironment (TME) of neuroblastoma most of these approaches fail (15–18).

Neuroblastoma tumors are infiltrated by diverse immune cells, including myeloid populations—such as tumor-associated macrophages (TAMs), neutrophils, dendritic cells, and myeloid-derived suppressor cells (MDSCs)—and lymphoid populations, including T cells and NK cells (16, 19). These cells interact to create a profoundly immunosuppressive microenvironment through myeloid recruitment, immune checkpoint expression, and cytokine-mediated inhibition of T and NK cells (2, 16, 17, 19–21). Among these myeloid populations, TAMs and MDSCs have recently emerged as central regulators of tumor progression and immune evasion in neuroblastoma (19, 22–26). While the role of TAMs in disease progression and therapeutic targeting has been previously reviewed (19), this article emphasizes the broader myeloid landscape of neuroblastoma, with a particular focus on myeloid-derived suppressor cells (MDSCs). We outline their origin and development, phenotypic and functional heterogeneity within neuroblastoma, mechanisms that drive their recruitment and immunosuppressive activity, their blockade of T-cell and NK-cell function, and current strategies to deplete or reprogram MDSCs to enhance anti-tumor immunity.

## 2 Myeloid landscape in neuroblastoma

**Figure 1:**
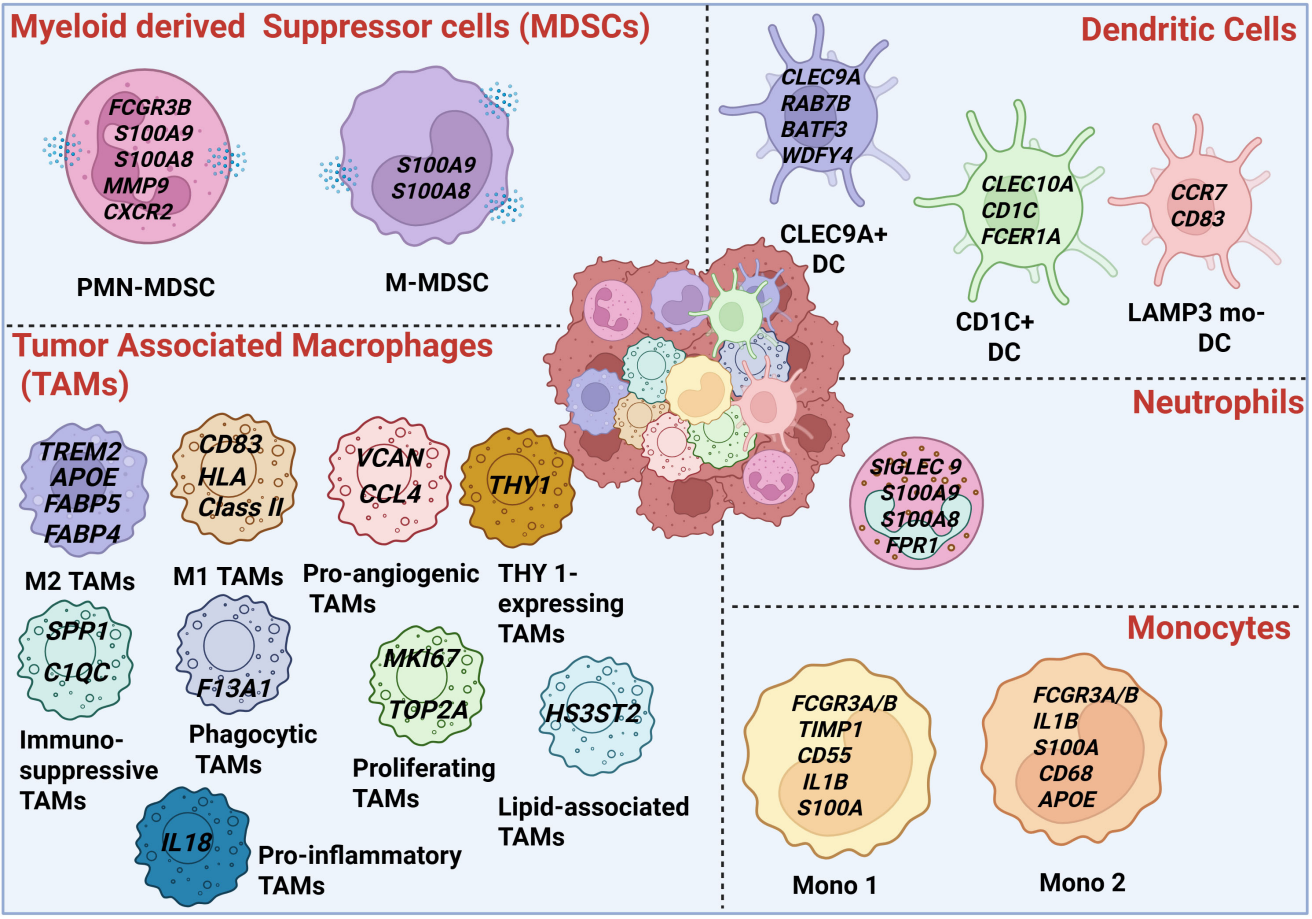
Overview of the myeloid landscape in neuroblastoma. The central panel illustrates a neuroblastoma tumor, highlighting the diverse myeloid populations within the tumor microenvironment. Specifically, it depicts the different subsets of MDSCs, tumor-associated macrophages (TAMs), neutrophils, and monocytes as identified from scRNA-seq datasets in neuroblastoma. This schematic provides a visual summary of the heterogeneity and composition of myeloid cells in these tumors. The figure was created with Biorender.com.

Recent studies have highlighted the critical role of myeloid cells in neuroblastoma progression (2, 19, 22, 24, 27, 28). Comprising monocytes, granulocytes (neutrophils and eosinophils), macrophages, dendritic cells, and MDSCs, these populations can promote either immunostimulation or immunosuppression and represent a major obstacle to effective treatment (16, 17, 21). MDSCs are immature myeloid cells that expand under conditions of stress, infection, or cancer progression (29). Instead of differentiating into mature macrophages or neutrophils, they remain in an immature state and give rise to two major subsets: monocytic MDSCs (M-MDSCs) and polymorphonuclear MDSCs (PMN-MDSCs), which are described in detail in subsequent sections of this review. This section discusses the roles of macrophages, neutrophils, and dendritic cells in driving neuroblastoma progression, with the various myeloid cell subsets identified in neuroblastoma tumors based on the scRNA-seq data shown in **Figure 1**.

### 2.1 Tumor-associated macrophages

Macrophages represent a key myeloid subset that drives neuroblastoma progression. Various tumor-derived factors recruit monocytes and M-MDSCs into the tumor microenvironment, where they subsequently differentiate into TAMs (26, 30–32). Shaped by environmental cues, these cells exhibit remarkable plasticity, with functions ranging from tumor-promoting (aka, M2 macrophages) to tumor-suppressing (aka, M1 macrophages) (19). With the advent of single-cell transcriptomics, it is now evident that macrophages within the NB TME exist in diverse phenotypic states, moving beyond the traditional M1/M2 paradigm (26, 32). Consistent with this, Verhoeven et al. analyzed 19 human neuroblastoma samples using scRNA-seq and identified four macrophage populations (Macro1–4) characterized by high *CD68* expression, as well as two monocyte clusters defined by key monocyte marker genes *CLEC7A, NLRP3, and BST1* (26). This study revealed that Macro-1 with macrophage-associated genes: *TREM2*, *FABP5*, and *FABP4* resemble M2 in phenotype. However Macro-2 with high expression of *CD83*, HLA class II genes, resemble M1 in phenotype. This study also identified two monocyte populations with elevated expressions of the pro-inflammatory cytokine *IL1B* and several inflammation-related *S100A* genes. Mono1 showed higher expression of *FCGR3A*, *FCGR3B*, *IFITM3*, *TIMP1*, *S100A4*, *S100A12*, and *CD55*, whereas Mono2 exhibited a mixed expression profile, including some Mono1 genes as well as macrophage-associated genes such as *CD68* and *APOE* (**Figure 1**). In another study by Costa etal, sc-RNA seq analysis on ten human patient samples found 4 different clusters and all clusters show high expression of *CD68* and *APOE* and resemble M2 macrophages (32). Another recent study has longitudinally profiled 22 patients with high-risk neuroblastoma before and after induction chemotherapy and found different macrophage clusters including proliferating state (*MKI67* and *TOP2A*), a pro-inflammatory state (*IL18*), two pro-angiogenic states (*CCL4* and *VCAN*), an immunosuppressive state (*C1QC* and *SPP1*), a tissue-resident state with the highest expression of a phagocytosis gene (*F13A1*), a lipid-associated state (*HS3ST2*) and an undefined state expressing *THY1* (23) (**Figure 1**). Interestingly, they identified an IL18^+^ macrophage population resembling M1-like macrophages, whose predominance correlated with improved treatment responses and reduced adverse clinical events. These findings strongly suggest that the presence of immunostimulatory macrophages may serve as a predictor of response to chemotherapy or immunotherapy in neuroblastoma. All the different subsets of monocytes and macrophages identified across various scRNA-seq datasets are detailed in **Figure 1**. Additionally, targeting TAMs has been shown to improve responses to immune checkpoint blockade or chemotherapy in preclinical models of neuroblastoma (24, 28). Importantly, spleen tyrosine kinase (SYK), has recently been shown to modulate macrophage responses (33–35). Inhibition of SYK using the FDA-approved inhibitor fostamatinib enhanced the efficacy of radiation and immunotherapy in neuroblastoma (24). The bone marrow, a key metastatic niche for neuroblastoma, harbors an immune microenvironment that remains poorly characterized. Recent single-cell transcriptomic and epigenomic profiling of bone marrow aspirates from patients across major neuroblastoma subtypes revealed distinct molecular and cellular features that shape tumor progression (36). These findings highlight the critical role of tumor–myeloid cell interactions in the metastatic niche and emphasize their potential as therapeutic targets to enhance immunotherapy efficacy.

### 2.2 Neutrophils

Neutrophils, also comprise another myeloid subset that has been documented in neuroblastoma by various studies (22, 37). However, it is inconclusive if its presence can be correlated with disease progression. Morandi et al. demonstrated that patients with localized neuroblastoma exhibited higher peripheral neutrophil counts than those with metastatic disease, and elevated neutrophil levels were associated with improved overall survival (OS) (38). A study by Zeng et al. also reported that increased neutrophil numbers correlate with improved survival (39). However, other studies did not find significant correlation between neutrophils (40) and survival or find that increased neutrophils are associated with worse survival (41, 42). Similar to the controversies regarding the association of neutrophils with disease progression, capturing these cells and distinguishing them from PMN-MDSCs has been challenging, particularly in single-cell RNA sequencing datasets. A recent study by Weinke et al. performed scRNA-seq on 10 pre- and 14 post-chemotherapy samples (including 5 paired samples) and identified an MDSC/tumor associated neutrophil (TAN) cluster characterized by high expression of *S100A8* and *S100A9*, which appears to differentiate from a cluster of undifferentiated monocytes across 24 tumors (25). In another recent scRNA-seq dataset, neutrophils were not readily detected, yet immunostaining for neutrophil elastase confirmed their presence (26). Costa et al. didn’t identify neutrophil population in the scRNA-seq dataset from the TH-MYCN mouse model (32). However they identified, PMN-MDSC cluster that exhibited elevated expression of *S100a8, S100a9, and Cxcr2*, genes typically associated with neutrophils along with high levels of *Mmp9* and *Il1β* (32). In another study, Martínez-Sanz and colleagues analyzed datasets from 500 neuroblastoma patient biopsies, comparing differentially expressed neuroblastoma mRNA transcripts with a neutrophil gene signature obtained from healthy donors, as described by Grassi et al. (43). They reported upregulation of neutrophil-related transcripts (*CGR3B*, *FPR1, S100A8/9, and SIGLEC9*) in both early- and late-stage neuroblastoma compared with healthy adrenal gland tissue (44). There is a lack of studies investigating the interaction of neutrophils with other components of the neuroblastoma tumor microenvironment. However, recent studies have shifted focus toward neutrophils, highlighting their role as immunosuppressive PMN-MDSCs that suppress immune cell function.

### 2.3 Dendritic cells

Dendritic cells (DCs) are key antigen-presenting cells that recognize tumor antigens and activate specific T cells (45). They comprise several subsets, including monocyte-derived DCs (moDCs), plasmacytoid DCs (pDCs), conventional DCs (cDC1 and cDC2), and Langerhans cells (46). The presence of DC in neuroblastoma has been reviewed before (47). scRNA-seq analysis of human neuroblastoma has identified three (DC) subpopulations: CLEC9A^+^ cells, CD1C^+^ cells, and mature LAMP3^+^ moDCs which express high levels of *CCR7* and *CD83* (26). CLEC9A^+^ DCs exhibited high expression of *CLEC9A*, *RAB7B*, *BATF3*, *WDFY4*, and *CADM1* and resemble cDC1, whereas CD1C^+^ DCs showed high levels of *CD1C*, *CLEC10A*, *FCER1A*, and HLA class II antigen-presenting genes and resemble cDC2. A recent study showed that DCs and natural killer (NK) cells positively correlate with T-cell infiltration in human neuroblastoma at both the transcriptional and protein levels, and their presence is associated with favorable prognosis (48). Another study found that tolerogenic dendritic cells are induced in the neuroblastoma TME, where they attenuate the antitumor effects of invariant NKT (iNKT) cells (49). This study demonstrated that supernatants from neuroblastoma cell lines impair DC maturation by blocking CD14 downregulation and CD1a upregulation, reduce IL-12 and TNF-α production, and increase IL-6 and IL-10 from monocytes. DCs exposed to these supernatants also fail to effectively stimulate iNKT cells, resulting in reduced IFN-γ production, which can be restored by IL-12. Various stromal and tumor-derived factors can impair DC function and development, and neuroblastoma-derived gangliosides are known to specifically inhibit DC activity (47).

## 3 Origin and development of MDSCs in neuroblastoma

**Figure 2:**
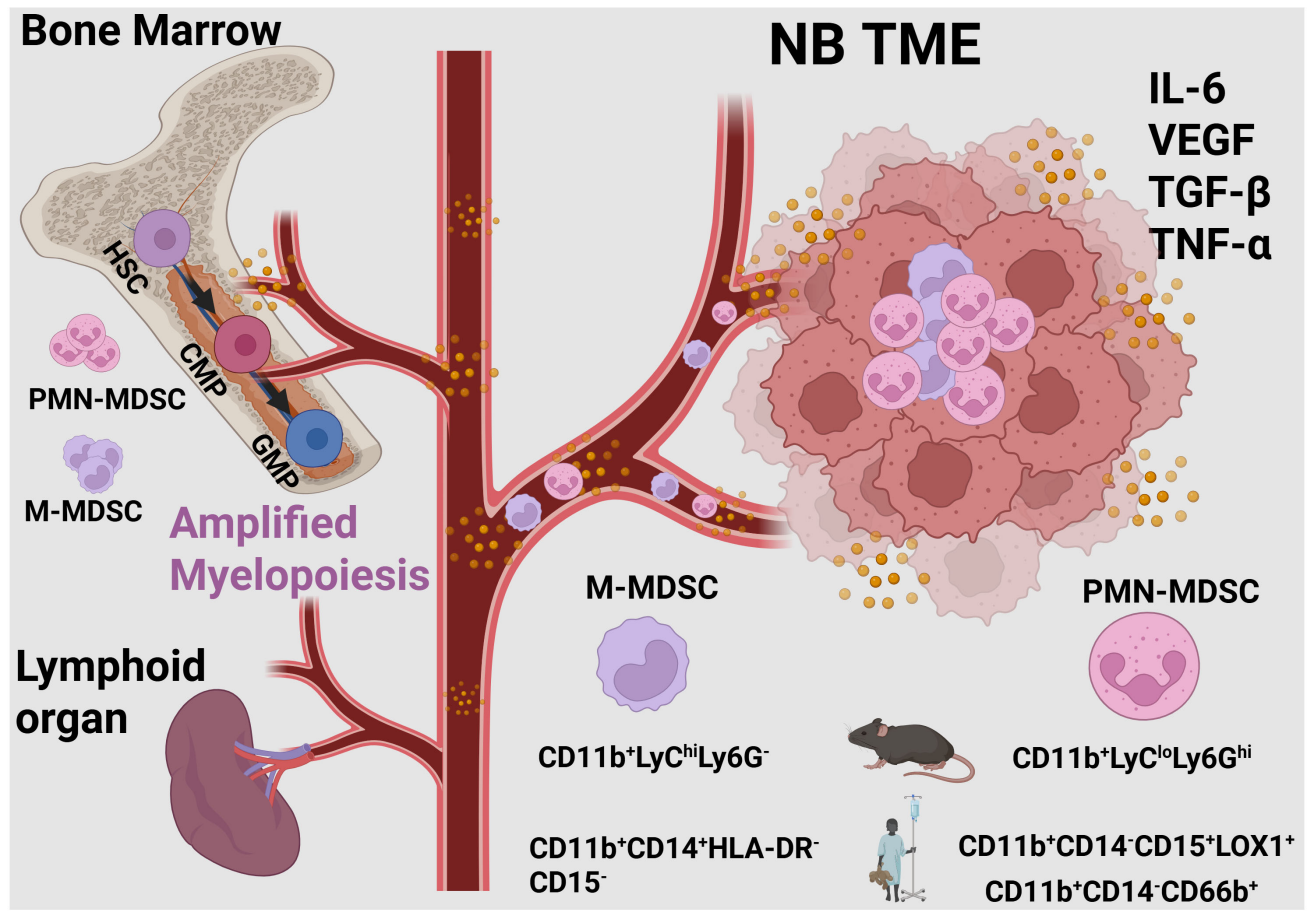
Differentiation of myeloid-derived suppressor cells in the NB TME. Various tumor-derived factors promote myelopoiesis, leading to the expansion and differentiation of myeloid cells into monocytic MDSCs (M-MDSCs) or polymorphonuclear MDSCs (PMN-MDSCs). M-MDSCs and PMN-MDSCs are identified based on distinct markers in both mice and humans, as illustrated. The figure was created with Biorender.com.

MDSCs are a heterogeneous population of myeloid cells that arise from hematopoietic stem cells (HSCs) through dysregulated myelopoiesis, progressing via common myeloid progenitors (CMPs) and granulocyte-monocyte progenitors (GMPs) (50) (**Figure 2**). In normal physiological conditions, CMPs are generated from HPCs within the bone marrow. CMPs subsequently migrate to secondary lymphoid organs, where they differentiate into monocytes and neutrophils (51). This pathway involves granulocyte-macrophage progenitors (GMP), myeloblasts (MB), and monocytic/dendritic cell precursors (MDP) as reviewed before (52).

In adult solid tumors, MDSCs primarily arise from chronic inflammation and sustained immune signaling, which drive emergency myelopoiesis and skew the differentiation of bone marrow progenitors toward immunosuppressive myeloid populations. Continuous exposure to tumor-derived factors—including vascular endothelial growth factor (VEGF), interleukin-1 (IL-1), interleukin-6 (IL-6), tumor necrosis factor-alpha (TNFα), transforming growth factor-beta (TGF-β), granulocyte-macrophage colony-stimulating factor (GM-CSF), granulocyte colony-stimulating factor (G-CSF), macrophage colony-stimulating factor (M-CSF), granulocyte colony stimulating factor, and S100 calcium-binding protein A9 (S100A9)—impairs myeloid cell maturation, promoting the development of immunosuppressive myeloid populations named as MDSCs (53). This process of myelopoiesis leads to a spectrum of immature myeloid cells, which are morphologically similar to monocytes or granulocytes but can be distinguished by the presence of specific cell surface markers (54).

In neuroblastoma, MDSC development is shaped by the tumor’s unique developmental and pediatric context, which differs from adult solid tumors. While adult tumors arise from epithelial tissues exposed to chronic inflammation and high mutational burden—conditions that drive emergency myelopoiesis—neuroblastoma originates from neural crest–derived sympathoadrenal progenitors, and its tumor microenvironment is relatively immune-cold and low in chronic inflammatory signals (16, 17, 21). In neuroblastoma, the factors driving MDSC accumulation remain poorly understood, although several studies have highlighted elevated levels of IL-6, VEGF, and TGF-β in the tumor microenvironment, which contribute to tumor aggressiveness (55–57). Analyses of serum cytokines in pediatric NB patients also show that IL-6 and TNF-α are significantly higher compared to healthy controls (57), suggesting these factors could influence MDSC development (58). Although these same cytokines drive MDSC expansion in adult tumors, their direct role in promoting MDSC accumulation in neuroblastoma has not been demonstrated, and the developmental context of NB may modulate their effects differently. Similarly, G-CSF and GM-CSF have been shown to enhance the cytotoxic activity of neutrophils against neuroblastoma cells (59); however, their potential role in promoting the generation or expansion of MDSCs within the NB TME has not been determined and is discussed further in this review. Collectively, these observations suggest that while multiple tumor- and stromal-derived factors modulate myeloid cell function, their precise contribution to MDSC accumulation in neuroblastoma remains to be elucidated.

## 4 Phenotypic and functional diversity of MDSC subsets in neuroblastoma

There are two main types of MDSCs: polymorphonuclear or granulocytic MDSCs (PMN or G-MDSCs), which resemble neutrophils; and monocytic MDSCs (M-MDSCs), which are similar to macrophages (50, 60, 61). In addition to M-MDSC and PMN-MDSC, another population of MDSCs, also known as “early-stage MDSCs” has also been identified in cancer patients and mouse models (50, 61, 62). A unique population of fibrocystic MDSCs has also been characterized in humans (63, 64).

In mice, MDSCs are primarily found in bone marrow, peripheral blood, spleen, and tumors. Murine MDSCs were initially defined as CD11b^+^Gr1^+^ (61). This broad definition has since been refined, with MDSC subsets further distinguished by their differential expression of Ly6C and Ly6G. Phenotypically, in mice, monocytes or M-MDSCs are defined as CD11b^+^Ly6C^hi^LY6G^-^ and PMN-MDSCs or neutrophils are characterized as CD11b^+^Ly6C^low^LY6G^hi^ (**Figure 2**). In humans, MDSCs are primarily observed in the blood, bone marrow, and tumors of various organs. MDSCs have also been detected in the peripheral blood of healthy infants, albeit at lower frequencies and with slightly greater antimicrobial activity than those observed in cancer patients (65). In humans, M-MDSCs, or monocytes, are defined as CD11b^+^HLA-DR^-^CD14^+^CD15^-^, while PMN-MDSCs, or neutrophils, are defined as CD11b^+^HLA-DR^-^CD14^-^CD15^+^/CD66b^+^. Because there are no well-defined phenotypic markers that reliably distinguish monocytes from M-MDSCs or neutrophils from PMN-MDSCs, they are often identified based on their immunosuppressive function (60, 61). Advances in transcriptomic and proteomic profiling have uncovered unique gene expression programs that distinguish diverse myeloid cell phenotypes in the TME (66–68). Recent studies have identified novel markers that differentiate monocytes from M-MDSCs and granulocytes from PMN-MDSCs (29). In humans, PMN-MDSCs share a similar phenotype with neutrophils but can be separated based on density, with PMN-MDSCs isolated at 1.077 g/ml and neutrophils at 1.2 g/ml. Furthermore, LOX-1 has been identified as a specific marker for PMN-MDSCs, enabling their detection in both blood and tumor samples from cancer patients (69).

A recent study provided valuable insights into the molecular characteristics and heterogeneity of MDSCs in human newborns by performing scRNA-seq on immature myeloid cells isolated from the peripheral blood of term infants, preterm infants, and adult controls (65). The study revealed that neonatal PMN-MDSCs comprise heterogeneous clusters representing a continuum of maturation, including PMN-MDSC precursors, classical PMN-MDSCs, late PMN-MDSCs, and mature neutrophils. Consistent with the diminished MDSC function observed in preterm infants, late PMN-MDSCs were more abundant in this group and exhibited reduced expression of genes associated with antimicrobial activity. Moreover, neonatal PMN-MDSCs displayed weaker immunosuppressive properties but stronger antibacterial functions compared to PMN-MDSCs from cancer patients (65).

Studies using neuroblastoma mouse models (NXS2 and NB9464) have shown a significant increase in CD11b^+^Gr1^+^ MDSCs in tumor-bearing mice compared to controls, with CD11b^+^Ly6G^+^Ly6C^low^ PMN-MDSCs being more abundant than CD11b^+^Ly6G⁻Ly6C^high^ M-MDSCs (27, 70). Similarly, elevated levels of MDSCs have been observed in the blood and spleen of tumor-bearing mice. Consistent with these findings, Costa et al. reported an increased abundance of CD11b^+^Ly6G^+^Ly6C^low^ PMN-MDSCs compared to CD11b^+^Ly6G⁻Ly6C^high^ M-MDSCs in TH-MYCN tumors (32). They further identified a PMN-MDSC population in the scRNA-seq dataset from the TH-MYCN mouse model. This cluster exhibited elevated expression of *S100a8, and S100a9*, genes typically associated with neutrophils, as well as increased expression of *Mmp9, Il1β* and *Arg2*, which are linked to immunosuppressive activity. These findings suggest that PMN-MDSCs represent a subset of neutrophils with immunosuppressive properties. Additionally, immunohistochemical quantification of *S100A8* was performed on these tumors to validate this observation. Another study by Tumino et al. demonstrated that CD45^+^Lin⁻HLA-DR⁻/^lo^CD33^+^CD11b^+^CD14⁻CD66b^+^ PMN-MDSCs with inhibitory activity are highly enriched in the peripheral blood of neuroblastoma patients with refractory or relapsed disease following GD2 CAR-T cell therapy (15). Interestingly, while circulating MDSC levels in the peripheral blood of cancer patients correlate with disease progression and serve as a prognostic indicator across multiple adult cancers (52, 71), in neuroblastoma they have not been linked to overall survival; however, Costa et al. has shown that patients with more aggressive MYCN-amplified tumors have been reported to harbor higher numbers of MDSCs compared to those with non–MYCN-amplified tumors, suggesting that enhanced immunosuppression contributes to their poorer clinical outcomes (32).

## 5 Mechanisms driving MDSC expansion and recruitment in the neuroblastoma tumor microenvironment

**Figure 3:**
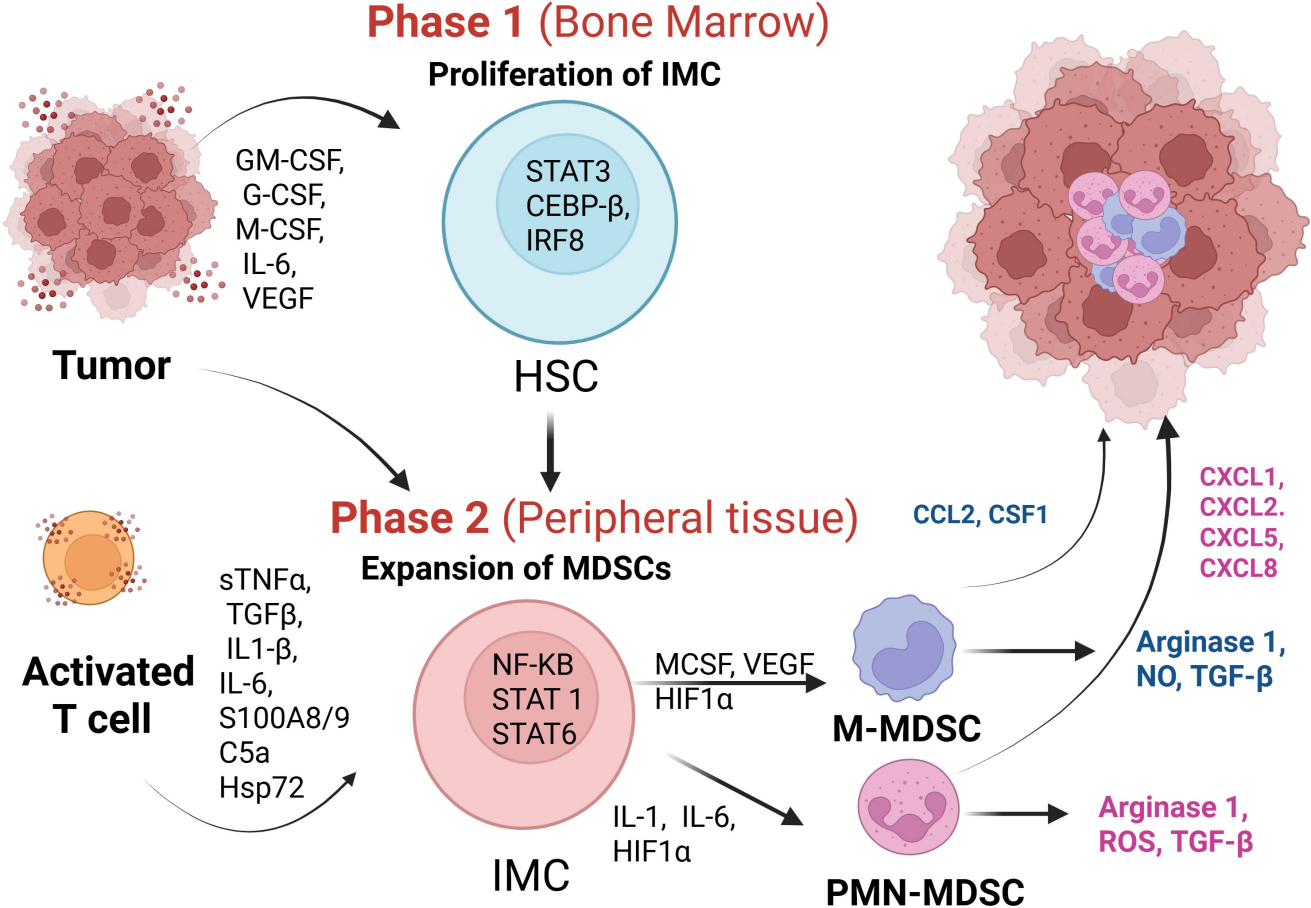
Mechanisms of MDSC generation and recruitment. In Phase 1, expansion signals triggered by factors such as M-CSF, GM-CSF, G-CSF, IL-6, and VEGF, mediated through STAT3, promote the proliferation and mobilization of immature myeloid cells from the bone marrow. In Phase 2, activation signals induced by stimuli including TNFα, TGFβ, IL-1β, and IL-6 drive the functional activation of M-MDSCs and PMN-MDSCs, leading to increased expression of ARG-1, NO, ROS, and immunosuppressive cytokines. Recruitment of M-MDSCs and PMN-MDSCs to the tumor microenvironment is guided by C-C and C-X-C chemokines. The figure was created with Biorender.com.

In adult tumors, MDSC generation is commonly explained by the two-signal model proposed by Gabrilovich et al. (72). In the first phase, tumor cell–derived factors such as GM-CSF, M-CSF, G-CSF, IL-6, and VEGF drive the expansion of MDSCs through emergency myelopoiesis (**Figure 3**). These signals promote the proliferation of immature myeloid cells, inhibit their differentiation, and mobilize them from the bone marrow. The process is primarily regulated by signal transducer and activator of transcription 3 (STAT3), CCAAT/enhancer-binding protein-β (C/EBPβ), and interferon regulatory factor 8 (IRF8). However, signals generated during the first phase are insufficient to induce the accumulation and activation of MDSCs. In the second phase, tumor- and T cell–derived cytokines (sTNFα, TGFβ, IL-1β, IL-6), additional factors such as prostaglandin E2 (PGE2), S100A8/9 proteins, complement component C5a, and exosome-associated Hsp72 further drive the differentiation of immature myeloid cells (iMCs) into MDSCs within peripheral tissues (54, 72–76). This effect is primarily mediated by the transcription factors nuclear factor kappa B (NF-κB), signal transducer and activator of transcription (STAT1) and STAT6. Elevated levels of M-CSF, VEGF, and hypoxia-inducible factor 1α (HIF1α) promote the development of M-MDSCs, whereas high levels of GM-CSF, IL-1, IL-6, adenosine, and HIF1α favor the development of PMN-MDSCs.

In contrast to adult tumors, which typically arise in the context of chronic inflammation, high mutational burden, and tissue injury that drive myeloid recruitment and activation, neuroblastoma originates from the malignant transformation of neural crest–derived cells during development, with myeloid recruitment largely governed by developmental programs rather than inflammatory signals. Neural crest–derived signals and oncogenic drivers such as MYCN play a central role in shaping the myeloid landscape in neuroblastoma, influencing both the differentiation and functional polarization of tumor-infiltrating myeloid cells. Consequently, MDSC accumulation and activity in neuroblastoma may not strictly follow the two-signal model established in adult tumors. While tumor-derived VEGF and IL-6 are elevated in neuroblastoma and correlate with poor prognosis (56, 77–80), direct evidence that these factors drive MDSC expansion in pediatric tumors is limited. Nevertheless, based on the two-signal framework, G-CSF and GM-CSF may synergize with tumor- and T cell–derived cytokines to promote MDSC expansion within the neuroblastoma tumor microenvironment. Supporting this possibility, studies in the NB9464 murine neuroblastoma model demonstrate that activated M-MDSCs and PMN-MDSCs upregulate ARG-1, nitric oxide (NO), reactive oxygen species (ROS), and immunosuppressive cytokines, collectively suppressing anti-tumor immune responses (27) (**Figure 3**).

The recruitment of M-MDSCs and PMN-MDSCs into the tumor microenvironment is regulated by mechanisms similar to those that govern monocyte and neutrophil trafficking (81). The recruitment of inflammatory monocytes and M-MDSCs in solid tumors is regulated by cancer cell–derived chemokines, primarily CCL2 and CCL5, which trigger a coordinated chemokine cascade (82). Metastatic neuroblastoma cells have been shown to express CCL2, a chemokine linked to high-risk disease (83). Functional studies indicate that recombinant CCL2 enhances tumor cell invasiveness, an effect that can be mitigated by blocking CCL2 with specific antibodies. MYCN amplification also shapes the immune landscape of neuroblastoma: MYCN represses interferon signaling and antigen presentation programs while also downregulating chemokines such as CCL2. As a result, MYCN-driven suppression of CCL2 limits the entry of CCR2^+^ antigen-presenting myeloid cells into the tumor, thereby reducing anti-tumor immune surveillance and potentially altering the balance between beneficial myeloid cell recruitment and MDSC accumulation (84). CSF1 also contributes to both the expansion and recruitment of MDSCs within the neuroblastoma tumor microenvironment (28). CSF1 signaling also induces immunosuppressive myeloid cells in neuroblastoma, and targeting this pathway can enhance immunotherapy.

CXCR2 plays a critical role in mediating neutrophil and PMN-MDSC recruitment to the tumor microenvironment through its interaction with CXC chemokine ligands, including CXCL1, CXCL2, CXCL5, and CXCL8. Elevated expression of these ligands in tumors promotes CXCR2-dependent trafficking of neutrophils and PMN-MDSCs, contributing to tumor progression, angiogenesis, and immunosuppression across multiple cancer types (81, 85). CXCL2/CXCR2 signaling is reported to play a critical role in the invasive ability of neuroblastoma cells co-cultured with macrophages (86). However, whether CXCR2 inhibition affects the infiltration or immunosuppressive function of PMN-MDSCs in neuroblastoma remains unknown and warrants further investigation. S100A8 and S100A9 are calcium-binding proteins that also play a crucial role in recruitment of neutrophils and PMN-MDSCs to pre-metastatic sites in solid tumors (50, 87–90). In neuroblastoma, S100A9 is considered a potential biomarker, and *in vitro* studies have shown that S100A9 overexpression enhances the proliferation, migration, and invasion of NB cells (91). However, no *in vivo* studies have demonstrated that targeting S100A9 can reduce PMN-MDSC infiltration in neuroblastoma tumors, and future studies are needed in this direction.

## 6 MDSC mediated inhibition of T cell and NK cell anti-tumor immunity

**Figure 4:**
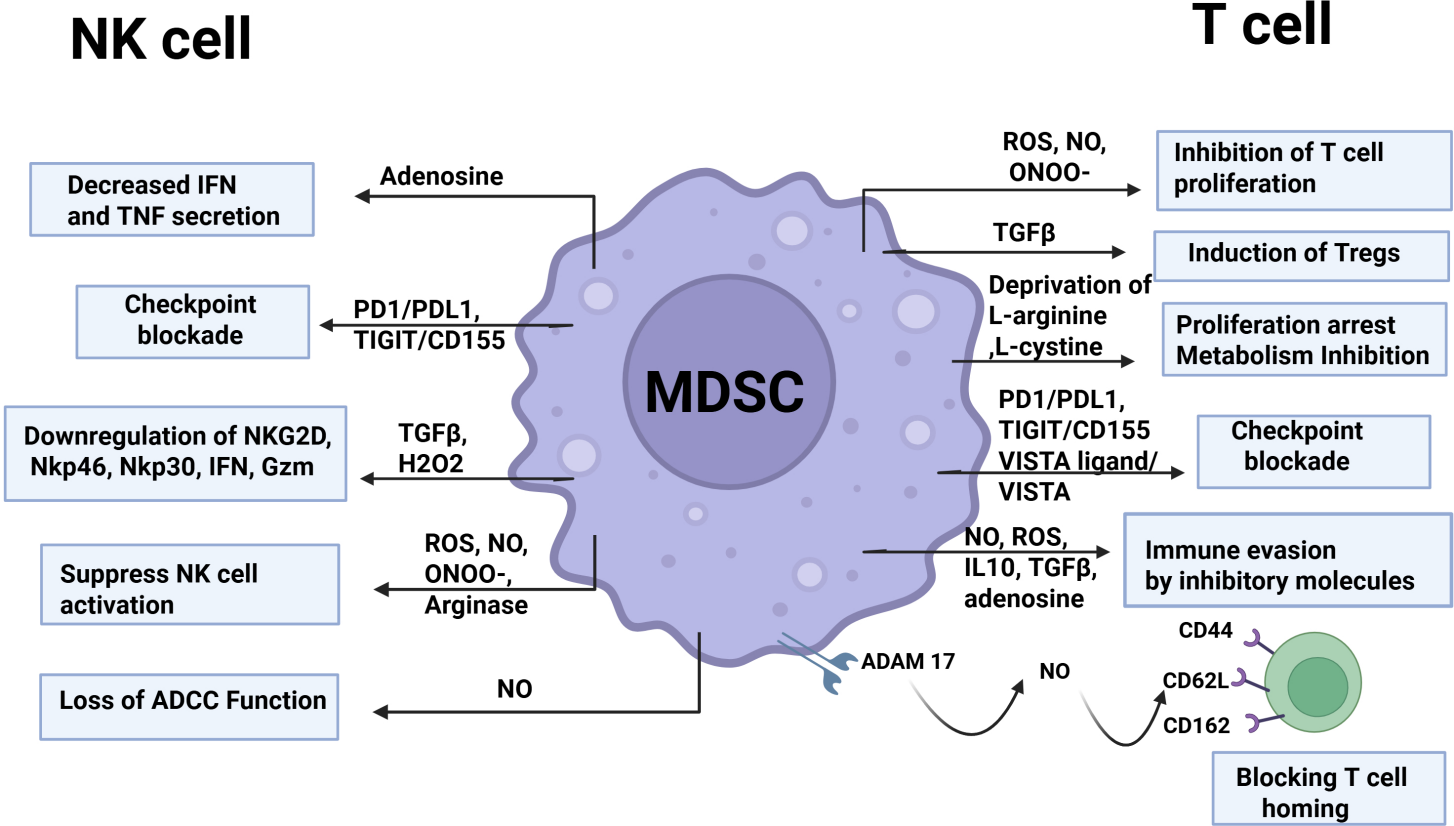
Schematic of strategies employed by MDSCs to inhibit T cell and NK cell function. MDSCs suppress T cell activity through multiple mechanisms, including the production of immunosuppressive molecules. They also impair NK cell function by secreting TGFβ, ROS, and NO.

MDSCs are key regulators of immune suppression and tumor progression (16, 27, 53, 92). They exert potent inhibitory effects on both T cells and natural killer (NK) cells (**Figure 4**). Although M-MDSCs and PMN-MDSCs share common immunosuppressive features—such as elevated arginase activity, S100A8/A9 expression, and STAT3 activation, they employ distinct mechanisms to modulate immune responses. PMN-MDSCs primarily mediate suppression through ARG-1, TGFβ, PGE2, and ROS, whereas M-MDSCs utilize NO, TGFβ, IL-10, and PD-L1 expression to inhibit T cell and NK cell activity (29).

### 6.1 Inhibition of T cell responses

MDSCs suppress T cell function through multiple mechanisms, including inhibition of lymphocyte trafficking, generation of reactive oxygen and nitrogen species, depletion of amino acids essential for T cell activity, expression of inhibitory immune checkpoint molecules, and expression of ectoenzymes that regulate adenosine metabolism as reviewed before (50, 52) (**Figure 4**). MDSCs inhibit T cell trafficking by downregulating key adhesion molecules. For instance, spleen MDSCs reduce L-selectin (CD62L) on CD4^+^ and CD8^+^ T cells via ADAM-17, impairing homing and antigen-dependent activation (54, 93). Additionally, NO from M-MDSCs decreases CD44, CD62L, and CD162 on T cells and lowers E-selectin on tumor vessels, collectively limiting T cell tumor infiltration (94, 95).

MDSCs also produce ROS and reactive nitrogen species (RNS), including NO, which impair T cell function (96). ROS, generated primarily via NADPH oxidase isoforms (NOX1–4), cause oxidative stress and nitration of TCR/CD8 molecules, disrupting TCR/MHC interactions (97). NO produced by iNOS in MDSCs further inhibits T cells by interfering with IL-2 signaling and promoting TCR nitration (98, 99), while combining with O2⁻ to form peroxynitrite (ONOO⁻), enhancing immunosuppression (100).

MDSCs also deplete amino acids essential for T cell metabolism, particularly L-arginine. L-arginine is metabolized by NOS isoforms into NO and L-citrulline, and by arginases into L-ornithine and urea, with L-ornithine further forming immunosuppressive polyamines. Tumor-derived factors and Th2 cytokines (IL-4, IL-10, IL-13) upregulate ARG-1 in MDSCs, while Th1 cytokines (IFNγ, TNFα) induce iNOS expression (101). MDSCs release ARG1 in extracellular microenvironment which resulted in reduced levels of L-arginine and inhibition of T cells. The subsequent deficiency of arginine results in the loss of CD3 zeta and the inhibition of T cell proliferation (102). A study by Ishfaq et al. demonstrated that M-MDSCs and PMN-MDSCs secrete NO and express high levels of ARG-1, TGF-β, and IDO, leading to the inhibition of CD4^+^ and CD8^+^ T cell proliferation in an *in vitro* model of neuroblastoma (27). This immunosuppressive effect can be reversed by blocking BTK signaling with ibrutinib.

MDSCs also suppress T cells through the expression of immune checkpoint molecules such as PD-L1, VISTA, CD155. PD-L1 is a key negative regulator of T cell function, and MDSCs induce T cell anergy via PD-1 interaction (92, 103, 104). Cassetta et al. demonstrated that in cancer patients, PDL1 expression was specifically observed in M-MDSCs and e-MDSCs (105). In tumor tissues obtained from patients with non-small cell lung cancer, tumor-associated PMN-MDSCs exhibit elevated expression of PDL1 compared to peripheral blood of same patients (106). PD-L1 expression on PMN-MDSCs was elevated in melanoma patients who failed to respond to ipilimumab compared to responders (107). PD-L1 expression has been found to be elevated in MDSCs and TAMs from both human and murine neuroblastoma (24, 27, 108). Moreover, one study reported that PD-L1 expression is further increased in patients treated with chemotherapy (108). Recent studies have shown that some other immune check point molecules such as VISTA, and CD155 have been reported to mediate immunosuppression (25, 109). A study has demonstrated that MDSCs in neuroblastoma tumors express CD155, with expression levels being higher in M-MDSCs compared to PMN-MDSCs (109). CD155 engages the inhibitory receptor TIGIT, leading to suppression of T cell and NK cell effector functions within the neuroblastoma tumor microenvironment (109).

T cells are also inhibited by adenosine, which is generated from extracellular ATP through the sequential actions of CD39 (E-NTPDase1) converting ATP to AMP and CD73 (ecto-5’-nucleotidase) converting AMP to adenosine. Adenosine suppresses naïve T cell priming and reduces effector molecules on activated T cells, including CD25, perforin, IFNγ, and TNFα (110). A study by Bianchi et al. has shown that the neuroblastoma TME contains high levels of extracellular ATP and adenosine, which influence immune regulation and tumor progression (70). This study found that extracellular ATP increases with tumor growth and that MDSCs, particularly M-MDSCs, express functional P2X7 receptors (P2X7R). Activation of P2X7R enhances M-MDSC immunosuppressive activity by upregulating ARG-1, TGF-β1, and ROS. This study reveals that the ATP/P2X7R axis within the ATP-rich neuroblastoma microenvironment promotes MDSC-mediated immune suppression and tumor progression.

### 6.2 Inhibition of NK cell responses

NK cells are innate immune cells that serve as the main effector cells in anti-GD2 immunotherapy but are largely immunosuppressed in the neuroblastoma tumor microenvironment due to tumor- or stroma-derived factors (111). Recent studies have highlighted that MDSCs also inhibit NK cell functions in neuroblastoma tumors (15, 112). MDSCs suppress NK cells through two main mechanisms: (a) direct cell-to-cell contact and (b) the release of soluble factors that impair NK cell cytotoxicity, cytokine production, and overall activation (52, 113). In contact-dependent inhibition, direct interactions between surface molecules on MDSCs and NK cells, such as inhibitory ligands or checkpoint molecules, can impair NK cell activation, reduce cytotoxic granule release, and limit cytokine production. In contact-independent inhibition, MDSCs secrete soluble factors—including ROS, NO, arginase-1, TGF-β, and immunosuppressive cytokines—that disrupt NK cell function by interfering with receptor signaling, metabolic pathways, and effector molecule expression.

Several preclinical studies in adult tumor models have shown that CD11b^+^Gr1^+^ MDSCs accumulate in the spleens of tumor-bearing mice, impairing NK cell cytotoxicity by downregulating activating receptors such as NKG2D and NKp30, and by reducing IFN-γ and perforin production (113–115). A recent study demonstrated that PMN-MDSCs preferentially expand in the peripheral blood of both murine and human neuroblastoma and impair NK cell function by downregulating the expression of NKG2D and NKp46 (112).

Studies in adult tumor–bearing mouse models have demonstrated that MDSC-mediated suppression of NK cell function requires direct cell–cell contact, with TGF-β acting as a key mediator of this inhibitory mechanism (115, 116). In a murine liver cancer model, membrane-bound TGFβ reduces NKG2D expression and IFN-γ production in NK cells, leading to impaired NK cell cytotoxicity (115). This study demonstrates that cell to cell contact between MDSC and NK cells is essential to induce NK cell anergy (115). Another study has shown that CXCR2+ MDSCs positively accumulate in the spleens of mice bearing head and neck tumors and suppress NK cell function through TGFβ and production of H_2_O_2_ (114). In neuroblastoma models, inhibition of TGF-βR1 signaling with the small-molecule inhibitor galunisertib decreased SMAD2 phosphorylation in both tumor cells and NK cells, restoring NK cytotoxic function (117). Notably, this blockade also enhanced NK-mediated antibody-dependent cellular cytotoxicity when combined with anti-GD2 therapy, highlighting TGF-β’s direct role in suppressing NK effector functions and revealing a therapeutic strategy to overcome MDSC-driven NK dysfunction.

NK cell activity can also be inhibited by factors produced by MDSCs, including NO, iNOS, peroxynitrite, ROS, and ARG1, which collectively promote immunosuppression and suppress NK cell activation. MDSC-mediated generation of ROS and ARG 1 also impairs the function of NK cells in cancer models *in vivo* (118). Moreover, MDSCs can secrete adenosine in TME by inducing the expression of CD39 in tumors (119). Adenosine suppresses NK cell cytotoxicity by reducing IFNγ and TNFα release (120) and also influences NK cell maturation (121). Stiff et al. reported that NO produced by MDSCs suppresses Fc-mediated NK cell functions, thereby reducing antibody-dependent cellular cytotoxicity (ADCC) and the production of IFNγ and TNFα (122). Inhibition of iNOS with the small-molecule inhibitor L-NIL restored NK cell function *in vitro* and *in vivo*, underscoring the pivotal role of NO in MDSC-mediated suppression (122). Although it remains to be determined whether MDSC-derived NO directly affects NK cell function in neuroblastoma, this mechanism could have significant implications, as the therapeutic efficacy of anti-GD2 antibody relies heavily on NK cell–mediated ADCC, which may be compromised by elevated NO levels in the tumor microenvironment. Although the direct impact of MDSCs on NK-mediated ADCC has not been specifically studied in neuroblastoma, recent evidence suggests that MDSCs contribute to poor responses to CAR-T therapy (15). In patients who failed to respond to GD2.CAR T-cell therapy, PMN-MDSCs were found to accumulate and were associated with impaired NK cell function.

In neuroblastoma, MDSCs also contribute to immune dysfunction by promoting the expression of checkpoint molecules that directly inhibit NK cell activity. Recent studies have identified PD-1/PD-L1 and TIGIT/CD155 as the most critical checkpoint pathways in this context (25, 109). A recent study has shown that anti-GD2 mAb–mediated ADCC strongly activates NK cells and induces effective tumor cell lysis, but it also triggers upregulation of PD-L1 on neuroblastoma cells and increases TIGIT and PD-1 expression on effector cells, especially NK cells (109). This study demonstrated that dual blockade of PD-L1 and TIGIT, in combination with dinutuximab beta, restored NK cell function, resulting in enhanced tumor cell killing and improved anti-tumor responses compared to blocking either checkpoint alone. Another study has shown that high TIGIT expression on NK cells is linked to their impaired function in neuroblastoma (25). This study further demonstrated that adding TIGIT and PD-L1 blockade to standard relapse treatment induced complete remission in a chemotherapy-resistant Th-ALK^F1174L^/MYCN 129/SvJ syngeneic model. Overall, these studies highlight that MDSCs suppress both T-cell and NK-cell responses, and that targeting or inhibiting MDSC function can enhance anti-tumor immunity in neuroblastoma.

## 7 Therapeutic strategies to target MDSCs in neuroblastoma

Multiple complementary strategies have been explored to overcome MDSC-mediated immunosuppression, including blocking their recruitment to tumors, depleting existing MDSCs, promoting their differentiation into mature non-suppressive cells, and inhibiting their immunosuppressive pathways (52, 123). These strategies have also been combined with other therapies, such as immune checkpoint inhibitors, to further mitigate MDSC-driven immune suppression (124). This section is divided into four parts, and the current strategies applied in neuroblastoma are summarized in **Table 1**.

**Table 1:** Various strategies used to target MDSCs in neuroblastoma.

Mechanism of action	Target	Treatment strategy	*In-vivo* models	Clinical trial number	Outcome	Clinical status
Inhibition of MDSC recruitment and trafficking	VEGF	Bevacizumab	HTLA-230, IMR-32 xenografts in Scid/Beige mice (125), Patient-Derived Xenograft (PDX) (126), SK-N-AS, IMR-32 and SH-SY5Y (127), NB1691 and CHLA-255 (128)	NCT01114555 NCT02308527	Increased T-cell infiltration	Bevacizumab is FDA approved and has been tested in combination with chemotherapy for relapsed or refractory neuroblastoma (129–131).
	Chemokine Receptors (CCL2, CCR2, CCL5)	Anti-CCL2 antibody + Etoposide	CHLA-255 Fluc NOD/SCID xenograft mouse model, COG-N-415X PDX (132)		Prolonged survival in residual disease	Anti-CCL2 therapies remain investigational, with no clinical trials conducted in pediatric neuroblastoma. Etoposide is FDA-approved and has been evaluated in a Phase 2 clinical trial for pediatric neuroblastoma (133).
	CSF1R	BLZ945	TH-MYCN neuroblastoma mouse Model (28), CHLA-255 Fluc NOD/SCID xenograft mouse model (134)		Reduce myeloid infiltration, reprogram macrophages to M1 phenotype, slower tumor growth, more effective response to PD-1/PD-L1 checkpoint blockade and chemotherapy	BLZ-945 has not been evaluated in clinical trials for pediatric neuroblastoma
Depletion of MDSCs	STAT1	Doxorubicin (low dose) or Dopamine + anti-GD2 Antibody	Syngeneic Neuro-2a tumors in Balb/c mice (135, 136)		Reduced MDSC infiltration. Increased tumor immunogenicity (increased CD8+ T-cell infiltration, HLA-I expression)	STAT1 inhibitors have not been tested in neuroblastoma clinical trials, whereas doxorubicin and anti-GD2 antibodies have been evaluated in such trials.
Inducing MDSC Differentiation	Immature MDSCs	All-trans Retinoic Acid (ATRA; Vitamin A Metabolite)	Kelly neuroblastoma xenografts in immunodeficient NOD.SCID.γc⁻/⁻ (NSG) mice (137)		Reduced MDSC population, increased CAR therapy efficacy.	
		All-trans Retinoic Acid (ATRA; Vitamin A Metabolite) with GM-CSF and 3F8 monoclonal antibodies		NCT01183429, NCT01183884, NCT01183416, NCT01183897, and NCT00969722	No results posted	ATRA has been tested in neuroblastoma across several trials, although none specifically targeted MDSC depletion.
		Polyphenon-E	TH-MYCN Neuroblastoma mouse model (138)		Reduced CD11b^+^ and Gr-1^+^ myeloid cell infiltration and population	Not tested in neuroblastoma clinical trials.
Blocking MDSC-mediated immunosuppression	JAK-STAT Pathway	AZD1480 or Ruxolitinib	NB-Tag spontaneous non-MYCN mouse model, syngeneic NBT2 mouse models (31)		Reduced TAM-mediated tumor growth with no significant effect on MDSCs.	Not tested in neuroblastoma clinical trials.
	HDAC	Vorinostat with anti-GD2	Syngeneic 9464D and 975A2 neuroblastoma tumors (139, 140)		Increased pro-inflammatory myeloid cell infiltration	Vorinostat is FDA-approved and has been tested in several neuroblastoma trials in combination with anti-GD2 therapy, radiation, and isotretinoin
		Vorinostat with, I-131 MIBG		NCT03332667, NCT01019850	No results posted	
		Vorinostat with isotretinoin		NCT01208454	No results posted	
	BTK	Ibrutinib	Syngeneic 9464D neuroblastoma tumors (27)		Increased anti- PD-L1 blockade efficacy	Ibrutinib is FDA-approved but has not been tested in clinical trials for neuroblastoma.
	TGFBR1	Galunisertib (LY2157299) monotherapy or with Mitoxantrone	CHLA-255-Fluc or CHLA-136-Fluc NOD/SCID xenograft mouse models (141), COG-N-415x patient-derived xenografts. Syngeneic 9464D and 975A2 neuroblastoma tumors (142)		Increased anti-GD2 and PD-L1 blockade efficacy	Not tested in neuroblastoma clinical trials.

The table summarizes each MDSC-targeting strategy, the molecular target, the *in vivo* model system used, and the observed outcomes.

### 7.1 Inhibition of MDSC recruitment and trafficking

M-MDSCs and PMN-MDSCs are actively recruited to the TME by various chemokines and growth factors (52). Therefore, targeting the signaling pathways and factors that drive MDSC recruitment and expansion could limit the accumulation of myeloid cells in the TME and suppress their tumor-promoting functions. Tumor cells serve as the primary source of VEGF within the TME (143). VEGF not only promotes angiogenesis but also functions as a chemoattractant for MDSCs. Although its direct role in MDSC expansion remains unclear, VEGF inhibition with agents such as bevacizumab, an anti-VEGF recombinant human mAb has been tested in neuroblastoma models, where it has been shown to enhance T-cell infiltration, potentially through direct or indirect effects on MDSC-mediated immunosuppression (125–128). Bevacizumab has also been evaluated in a recent phase II trial in combination with temozolomide or irinotecan-temozolomide (IT) or topotecan-temozolomide (TTo) in children with relapsed or refractory neuroblastoma (NCT02308527), where addition of bevacizumab has been shown to improve overall response rate (ORR) and progression-free survival (PFS) (129). In another trial combining bevacizumab with irinotecan and temozolomide for relapsed and refractory neuroblastoma (NCT01114555), the addition of bevacizumab did not improve response rates compared to historical data for irinotecan-temozolomide alone (130).

S100A8 and S100A9 are calcium-binding proteins that play a crucial role in the accumulation of MDSCs in the TME (50, 87–90). Inhibition of S100A8/A9 has been reported to reduce the accumulation of MDSCs in various mouse tumor models (87, 89). Tasquinimod is in clinical development for the treatment of prostate cancer and other cancers and is identified to inhibit the binding of S100A9 proteins to TLR4 and RAGE receptors (144). In neuroblastoma, S100A9 has been identified as a potential biomarker, with *in vitro* studies demonstrating that its overexpression promotes NB cell proliferation, migration, and invasion (91). However, tasquinimod, which has been evaluated in adult solid tumors, has not yet been tested in neuroblastoma and could be explored in future studies.

Chemokine receptors play a central role in directing the migration of MDSCs to the tumor site (145). M-MDSCs mainly express C-C motif chemokine receptors (CCR2) and are recruited in tumors expressing chemokines, CCL2 and CCL5 (146). Multiple studies have demonstrated that blocking the CCL2/CCR2 axis, either alone or in combination with immunotherapy or targeted therapy, reduces intratumoral MDSC accumulation and enhances antitumor efficacy in various preclinical mouse models (147–149). Metastatic neuroblastoma cells express CCL2, a chemokine associated with high-risk disease, and functional studies have shown that recombinant CCL2 enhances tumor cell invasiveness—an effect that can be reversed by blocking CCL2 with specific antibodies (83). A recent study demonstrated that combining an anti-CCL2 antibody with etoposide prolongs survival in a minimal-residual disease model of neuroblastoma (132).

The chemokine receptor CCR5 is crucial for MDSC recruitment through its ligands CCL3, CCL4, and CCL5. In melanoma, CCR5 blockade has been shown to reduce MDSC infiltration and improve survival (150); similarly, inhibiting CCR5 suppresses tumor growth and metastasis in breast, pancreatic, colorectal, and prostate cancers. (151–154). However, the role of CCR5 blockade in neuroblastoma has not yet been explored.

Inhibition of CSF1R signaling can also reduce MDSC recruitment to the tumor microenvironment. Binding of its ligand, CSF1, to CSF1R drives the differentiation and expansion of myeloid cells. Notably, CSF1R expression is elevated in neuroblastoma tumors (28). In multiple solid tumor models, pharmacological blockade of CSF-1R using monoclonal antibodies or small-molecule inhibitors have been shown to reduce myeloid cell infiltration and reprogram macrophages toward a more antitumor phenotype (155). In neuroblastoma, this strategy has been tested in the TH-MYCN model, where CSF-1R inhibition not only slowed tumor growth but also enhanced the efficacy of PD-1/PD-L1 checkpoint blockade (28). Furthermore, studies in human xenograft models demonstrated that depletion of suppressive myeloid cells via CSF-1R inhibition improved chemotherapy responses, even in T cell–deficient settings (134).

CXCR1/2 signaling is upregulated in PMN-MDSCs and neutrophils; therefore, blocking CXCR2 signaling, either alone or in combination with anti-PD-1 antibody, markedly enhances T cell responses in both mouse models and cancer patients (146, 156). Several studies have shown that CXCR2 deletion or inhibition with SX-682, reparixin, and AZD-5069 reduces the recruitment of PMN-MDSCs *in vivo* in pancreatic, lung, head and neck, and other cancer models (114, 157, 158). However, CXCR2 inhibitors have not been evaluated in neuroblastoma models, and the impact of targeting PMN-MDSC or neutrophil recruitment in this context remains unexplored.

### 7.2 Depletion of MDSCs

Chemotherapy drugs, such as gemcitabine, 5-Fluorouracil, docetaxel, oxaliplatin, paclitaxel, and doxorubicin, exerts favorable effects by depleting MDSCs, increasing the efficacy of immune therapies, and enhancing the anti-tumor activity of T cells and NK cells in adult solid tumors (159–165). Doxorubicin has shown significant immunomodulatory effects in neuroblastoma. Xu et al. reported that low-dose administration of doxorubicin or dopamine in neuroblastoma-bearing mice selectively depleted MDSCs. In the Neuro-2a model, doxorubicin at 2.5 mg/kg markedly reduced MDSC accumulation within the tumor microenvironment while avoiding the systemic toxicity associated with higher doses (135). Moreover, low-dose doxorubicin enhanced tumor immunogenicity by upregulating HLA-I expression and increasing CD8^+^ T cell infiltration. When combined with anti-GD2 antibody or antigen-specific cytotoxic T lymphocytes (CTLs), doxorubicin synergistically enhanced cytokine production, boosted perforin and granzyme release, and restored effector T cell function. Ultimately, low-dose doxorubicin enhanced the efficacy of immunotherapy in neuroblastoma by inhibiting tumor growth. Building on these findings, another study demonstrated that pharmacological depletion of MDSCs with low-dose doxorubicin reprograms the STAT signaling network by reducing STAT1 activation while enhancing the phosphorylation of STAT3, STAT5, and STAT6 (136). This shift impaired MDSC survival and expansion and reduced the T_reg_ population. Consequently, TAM polarization toward the immunosuppressive phenotype was limited, leading to decreased production of immunosuppressive mediators such as Arg-1, iNOS, and ROS. As a result, tumor-infiltrating T cells increased in number and exhibited lower PD-1 expression, ultimately slowing tumor progression.

### 7.3 Inducing MDSC differentiation

Promoting the differentiation of immature myeloid cells is another strategy used to reduce MDSC numbers in both murine tumor models and cancer patients. Multiple studies have demonstrated that vitamins A, D3, and E decrease the population of immature MDSCs and enhance T cell–mediated anti-tumor responses in murine models as well as in patients with head and neck cancer (166, 167). All-trans retinoic acid (ATRA), a metabolite of vitamin A, promotes the differentiation of MDSCs into mature myeloid cells (168–170). ATRA has been reported to induce the differentiation of mature antigen-presenting precursor cells, resulting in the modulation of T cell responses in both murine models and various human cancers (137, 169, 171). Several Phase II clinical trials are evaluating ATRA in combination with GM-CSF and 3F8 monoclonal antibody for neuroblastoma patients (NCT01183429, NCT01183884, NCT01183416, NCT01183897, and NCT00969722). ATRA-mediated reduction of MDSCs has been shown to enhance the efficacy of CAR therapy in neuroblastoma (137). Polyphenon E, a green tea catechin formulation, demonstrated potent anticancer activity in neuroblastoma by driving the differentiation and inactivation of MDSCs (138). In TH-MYCN transgenic mice, oral administration of Polyphenon E reduced tumor incidence, with ~50% of treated mice remaining tumor free at 8 months compared to 100% tumor penetrance in controls. Histological analysis of Polyphenon E–treated tumors revealed a marked reduction in CD11b^+^ and Gr-1^+^ myeloid cell infiltration, while flow cytometry confirmed decreased CD11b^+^/Gr-1^+^ MDSC populations in lymphoid organs. Mechanistically, Polyphenon E impaired MDSC motility and promoted their differentiation toward mature neutrophilic phenotypes through activation of the 67-kDa laminin receptor and induction of G-CSF.

### 7.4 Blocking MDSC-mediated immunosuppression

Modulating the immunosuppressive mechanisms utilized by MDSCs has been shown to enhance the cytotoxic activity of T and NK cells. The STAT family of transcription factors, particularly STAT3, is a key regulator of MDSC accumulation and expansion within tumors (66, 172). Accordingly, pharmacological inhibition of STAT3 using small-molecule inhibitors or curcumin has been shown to block the suppressive functions of MDSCs in multiple preclinical mouse models (173–175). Targeting STAT3 with oligonucleotide decoys (AZD9150/Danvatirsen) or siRNA, alone or alongside immune checkpoint inhibitors, effectively reduced granulocytic MDSCs in preclinical studies and Phase I/II trials (176–178). Histone deacetylase (HDAC) inhibitors have been shown to attenuate MDSC-mediated immunosuppression while enhancing the cytotoxic activity of T cells and NK cells (179, 180). Entinostat, an HDAC1 inhibitor, has been reported to potentiate T and NK cell function through suppression of MDSC-mediated immunosuppression (181, 182). Entinostat has been evaluated in neuroblastoma, and pretreatment of tumor cells with this HDAC inhibitor was found to enhance the cytotoxic activity of tumor-specific T cells *in vitro*, coinciding with increased expression of molecules that promote T cell–mediated killing (183). Another HDAC inhibitor, vorinostat, synergizes with anti-GD2 monoclonal antibody therapy to suppress neuroblastoma tumor growth (139). Vorinostat modulated the tumor microenvironment to favor antibody-mediated responses by increasing macrophages with high Fc receptor expression while reducing both the number and suppressive function of MDSCs. Another study demonstrated that combination therapy with anti-GD2 antibody and vorinostat is highly effective in an aggressive orthotopic neuroblastoma model (140). The treatment was associated with increased infiltration of myeloid cells, including macrophages, which displayed elevated MHC class II and Fc receptor expression. Several clinical trials have been completed or are currently ongoing to evaluate vorinostat in combination with therapies such as I-131 MIBG (NCT03332667, NCT01019850) and isotretinoin (NCT01208454) (**Table 1**).

Ibrutinib, a BTK inhibitor, can also target immunosuppressive MDSCs within the neuroblastoma tumor microenvironment. A study by Ishfaq et al. has shown that inhibiting BTK signaling with ibrutinib reduces MDSC immunosuppressive function and restores T cell–mediated anti-tumor immunity (27). Ibrutinib also enhances anti–PD-L1 immunotherapy by overcoming MDSC-driven immune suppression.

Small-molecule ARG-1 inhibitors have been reported to reduce iNOS and COX-2 expression and to modulate the immunosuppressive functions of MDSCs (184, 185). ARG-1 inhibition with CB-1158 reduced MDSC recruitment to the tumor microenvironment, enhanced tumor-infiltrating T and NK cell populations, and decreased tumor burden in preclinical models (184). In a Phase I clinical trial, the combination of CB-1158 and pembrolizumab was well tolerated in patients with advanced and metastatic tumors (NCT02903914). A recent study demonstrated that both murine and human neuroblastoma tumor cells suppress T cell proliferation through increased arginase activity (186). The study further showed that neuroblastoma-derived arginase activity impairs the proliferation and cytotoxicity of NY-ESO-1–specific TCR and GD2-specific CAR-engineered T cells. High arginase II expression is associated with poor survival in neuroblastoma patients. However, arginase I inhibitors have not yet been tested in preclinical neuroblastoma models and warrant further investigation.

CD39 and CD73, the key ectonucleotidases in the purinergic signaling pathway, convert extracellular ATP into adenosine, thereby creating an immunosuppressive microenvironment that inhibits T and NK cell activity (70). Elevated extracellular ATP can also activate P2X7 receptors, which are highly expressed on M-MDSCs in neuroblastoma. A study by Bianchi et al. showed that P2X7 signaling enhanced ARG-1, ROS, and TGF-β1 production in M-MDSCs without inducing cytotoxicity, thereby promoting potent immunosuppressive activity (70). Genetic deletion of P2X7 disrupted this pathway, reduced MDSC-mediated immunosuppression, and impaired tumor growth. Together, these findings suggest that the CD39/CD73–adenosine axis, in concert with P2X7 signaling, amplifies MDSC function and contributes to immune evasion in neuroblastoma. Targeting this interconnected purinergic network may thus represent an effective strategy to reprogram MDSCs and restore anti-tumor immunity.

Inhibition of TGFβ signaling has also shown promising results in reducing MDSC-mediated immunosuppression in preclinical models and clinical studies (117, 187–189). Galunisertib (LY2157299) is a small molecule that inhibits the kinase activity of TGFβR1 and has been evaluated in various clinical trials in adult solid tumors. TGF-β signaling is known to promote immunosuppression in neuroblastoma tumors, and its inhibition can enhance T and NK cell function by directly or indirectly modulating MDSCs (117, 141, 142, 190). TGF-β blockade has also been reported to enhance the efficacy of anti-GD2 therapy or anti-PD-L1 antibody treatment in neuroblastoma (117, 142).

## 8 Targeting ferroptosis to reprogram MDSCs in neuroblastoma

Ferroptosis is an iron-dependent form of regulated cell death characterized by the accumulation of lipid peroxides, distinct from apoptosis or necroptosis (191). In neuroblastoma, recent studies suggest that tumor cells are susceptible to ferroptotic cell death, particularly under conditions of oxidative stress or disruption of glutathione metabolism (192–194). Targeting ferroptosis has emerged as a potential therapeutic strategy in cancers resistant to apoptosis, including neuroblastoma (195). Unlike apoptosis, ferroptosis exploits the high iron demand and redox imbalance of tumor cells, rendering them particularly susceptible to ferroptotic inducers. A recent study identified the CDC27–ODC1 axis as a key regulator of ferroptosis in neuroblastoma. CDC27 enhanced tumor proliferation, metastasis, and sphere formation, while its knockdown reduced ODC1 expression and inhibited tumor growth (196). Notably, CDC27 overexpression sensitized neuroblastoma cells to ferroptosis, whereas ODC1 knockdown reversed this effect. RNA sequencing revealed that CDC27 downregulated SLC7A11, a component of the cystine/glutamate antiporter, leading to reduced cystine uptake, impaired glutathione synthesis, and increased lipid peroxidation. Importantly, the functional consequences of ferroptosis are highly cell-type dependent. While ferroptosis induction is beneficial in tumor cells, where it promotes tumor cell death and can enhance anti-tumor immunity, recent work indicates that PMN-MDSCs can undergo spontaneous or stress-induced ferroptosis that releases oxidized lipids (197). These lipid mediators suppress T-cell and NK cell activity, reinforcing the immunosuppressive tumor microenvironment and limiting the overall therapeutic benefit. Inhibition of ferroptosis with liproxstatin-1 preserved PMN-MDSC viability while abrogating their immunosuppressive activity, synergizing with immune checkpoint blockade. These findings highlight that effective therapeutic strategies may require selective modulation of ferroptosis: enhancing ferroptotic sensitivity in tumor cells to induce cytotoxicity, while limiting lipid peroxidation in myeloid populations to prevent immunosuppression. This could be achieved by carefully adjusting doses or using combination therapies that exploit tumor cells’ higher iron levels and oxidative stress to make them more sensitive to ferroptosis, while simultaneously depleting or reprogramming suppressive myeloid cells to prevent their immunosuppressive effects. Such approaches could include pairing ferroptosis inducers with MDSC-targeted therapies, immune checkpoint blockade, or agents that shift myeloid populations toward a pro-inflammatory, anti-tumor phenotype.

## 9 Balancing neutrophil activation and MDSC expansion: clinical considerations

G-CSF and GM-CSF are central to neuroblastoma therapy, serving both as essential supportive care cytokines during chemotherapy and as immune-enhancing agents during immunotherapy (198). G-CSF and GM-CSF are commonly administered to neuroblastoma patients to prevent chemotherapy-induced neutropenia and enhance host defense. During induction, G-CSF is routinely used to mitigate neutropenia and support the delivery of planned treatment intensity. In the randomized European HR-NBL1 trial, prophylactic G-CSF administered during rapid COJEC induction (cisplatin, vincristine, carboplatin, etoposide, and cyclophosphamide given on an accelerated schedule) significantly reduced febrile neutropenia and improved overall treatment deliverability (199). In addition, colony-stimulating factors are also required for hematopoietic stem cell mobilization before myeloablative consolidation, facilitating the reinfusion of collected progenitors to restore hematopoiesis (200). During the immunotherapy phase of treatment, cytokines such as GM-CSF assume an active immune-modulating role rather than serving solely as supportive care. GM-CSF is intentionally incorporated into anti-GD2 regimens to potentiate antibody-dependent cellular cytotoxicity (ADCC), thereby enhancing the therapeutic activity of effector cells (6, 8). The Children’s Oncology Group demonstrated this principle in a landmark trial showing that the combination of dinutuximab with GM-CSF and interleukin-2 significantly improved survival in patients with high-risk neuroblastoma, underscoring the clinical value of cytokine-driven immune amplification (6). Beyond immune modulation, G-CSF can also act directly on neuroblastoma cells. A subset of tumor cells has been reported to express the G-CSF receptor, CD114, and exhibits tumor initiating stem like properties (201). In these cells, G-CSF selectively activates STAT3 signaling, leading to expansion of the CD114-positive population and promoting tumor growth and metastasis *in vivo* (202). These observations raise the possibility that administration during minimal residual disease may preferentially expand aggressive tumor cell populations. In addition, G-CSF and GM-CSF have been implicated in driving the expansion and mobilization of MDSCs in multiple adult solid tumors, and likely exert similar effects in neuroblastoma, raising concerns about unintended immunosuppressive consequences within the TME. The balance between activating anti-tumor neutrophils and promoting immunosuppressive MDSCs is not yet fully understood in neuroblastoma, creating a critical clinical dilemma. Timing and context of administration may be key determinants of outcome. For example, delivering G-CSF or GM-CSF concurrently with immunotherapies could potentially blunt therapeutic efficacy if MDSC expansion predominates. Conversely, careful scheduling may allow exploitation of neutrophil-mediated cytotoxicity while minimizing immunosuppressive consequences. Future studies should aim to define the kinetics of MDSC generation following cytokine administration, identify biomarkers predicting risk of immunosuppression, and determine optimal integration of G-/GM-CSF with emerging immunotherapeutic strategies. Addressing this balance is critical for maximizing clinical benefit while minimizing potential counterproductive effects in neuroblastoma patients.

## 10 Conclusion

Myeloid cells have recently emerged as important targets in neuroblastoma, similar to their role in adult tumors (2, 19, 22, 24, 27, 28). Among these, macrophages and MDSCs are abundantly infiltrated in neuroblastoma tumors (24, 27). Several studies have demonstrated that these cells are highly immunosuppressive and dampen both T-cell and NK-cell responses (16, 17, 21). Macrophages, now recognized to exist beyond the traditional M1/M2 spectrum, display several phenotypes within neuroblastoma tumors. The presence of immunostimulatory macrophages has been associated with a favorable response to chemotherapy (23). The precise role of neutrophils in neuroblastoma progression remains a subject of debate. Their short lifespan, transcriptional similarity to PMN-MDSCs, and underrepresentation in scRNA-seq datasets make it challenging to accurately define their functional contribution within the tumor microenvironment. Several studies have identified the presence of MDSCs in neuroblastoma tumors (15, 27). While tumor-derived factors driving myelopoiesis are well characterized in several cancers, their roles in neuroblastoma remain poorly defined. Factors such as VEGF, IL-6, and TGF-β are known to promote neuroblastoma progression; however, whether they contribute to MDSC expansion has not been explored. Moreover, the contribution of G-CSF and GM-CSF to neuroblastoma progression remains unclear, particularly given their potential roles in modulating ADCC responses. Numerous studies have shown that PMN-MDSCs are more abundant than M-MDSCs in tumors and possess stronger intrinsic immunosuppressive activity (27, 32). Interestingly, while numerous MDSC-targeting strategies have been explored in adult tumors (52), very few have been tested in neuroblastoma. Approaches such as targeting VEGF or inducing MDSC differentiation with ATRA have shown promise, yet their direct effects on MDSCs in neuroblastoma remain poorly understood. In addition, agents like the HDAC inhibitor vorinostat have demonstrated encouraging results and are being evaluated in combination with I-131 MIBG and isotretinoin in clinical trials. However, many other MDSC-targeting agents remain unexplored in this disease. Given that MDSCs suppress NK and T cell function—both critical for effective anti-tumor immunity—combining MDSC-targeting strategies with NK cell– or T cell–based immunotherapies may represent a particularly promising approach for improving neuroblastoma outcomes.
